# 

**Published:** 1912-06-01

**Authors:** 


### Buildings Contemplated and in Progress.

BRIGHTON.—A Manlove-Alliott "oval" type steam disinfector is to be installed in the Brighton Guardians' premises at a cost of £288 10s.

BROMLEY (KENT).—Mr. Francis J. Smith, F.R.I.B.A., of London, is architect for projected alterations to the workhouse at Farnborough, including new receiving wards, etc. Tenders are invited.

BROMLEY (MIDDLESEX).—Additions and alterations are projected at the District Sick Mental Hospital, Devons Road, Bromley, E. Particulars may be had from the architects, Messrs. J. and W. Clarkson, 136 High Stree, Poplar, E. The contract contains a fair wages clause.

BUCKNALL.—The Stoke-on-Trent and Stoke Rural Joint Hospital Board have received sanction to borrow £6,724 for the purpose of extending the hospital. A tender of £2,920 has been accepted for a new heating system. The Local Government Board has also sanctioned a loan of £633 for the purchase of furniture.

CHANDLERSFORD.—Mr. W. Wallace Gandy, the Council's surveyor, is responsible for the design of the Infectious Hospital at Fryom Hill for the Eastleigh and Bishopstoke Urban Council. A tender of £5,060 has been accepted.

CLATTERBRIDGE (CHESHIRE).—Messrs. Davies and Sons, architects, of Chester, are issuing tender forms, etc., for various works at the Clatterbridge Workhouse. The scheme includes the erection of a master's house, a children's home, and a remodelling of the laundry block.

CROYDON.—The Croydon Town Council have accepted a tender of £1,710 for the erection of a new boiler-house. The work is under the charge of the Council's surveyor, Mr. Carter, who is responsible for the recent extensions and observation pavilion.

ENDERBY.—Alterations are projected at the Blaby Union Workhouse including the erection of new sanitary annexes. Mr. W. M. Cowdell, F.R.I.B.A., 12 Grey Friars, Leicester, is the architect, and particulars of tender may be had from him on deposit of two guineas.

KINGSTON-UPON-HULL.—Mr. T. Beecroft Atkinson, of Hull, is architect for a proposed infirmary at the workhouse in Anlaby Road, Hull. Tenders are invited.

LONDON.—The Metropolitan Asylums Board have appointed Messrs. W. T. Farthing and Son as quantity surveyors in connection with projected works at Darenth Asylum on a basis of 2½ per cent. on the accepted tender.

RADCLIFFE.—The Notts County Council have provisionally accepted a tender of £22,167 for the extension of the asylum. Instructions have been given for the preparation of plans for twelve cottages for staff.

RIBCHESTER.—At a meeting of the Ribchester Guardians the names of two architects were submitted and voted upon for the designing of a new workhouse. Mr. F. Howorth was appointed.

SANATORIA, NEAR CONSTANTINOPLE.—The *Zentral-Anzeiger* (Vienna) states that Mr. S. Haimovici, of Bucharest, has formed a company with a capital of £152,000, to be increased to £200,000, for the purpose of constructing and working two open-air sanatoria at Cury-Salova, which is two hours' journey from Constantinople.

SHEFFIELD.—Mr. A. F. Watson, Sheffield, is architect for a new operating theatre at the Ecclesall-Bierlow Workhouse. Tenders are now being considered.

SOUTH AFRICA.—It is reported that the estimates for loan expenditure 1912-13 for the Union of South Africa include two provisions for hospitals, one of £30,000 and one of £68,000.

ST. AUSTELL.—The female side of the Guardians' infirmary at the workhouse is to be improved at an estimated cost of £2,000.

SWITHLAND (LEICESTERSHIRE).—Messrs. Seale and Riley are architects for the Female Convalescent Home which has just been completed. The cost of the buildings was £17,000.

THORNTON HEATH (SURREY).—The Croydon Union invite tenders for the supply and erection of a Cornish boiler, 16 feet by 5 feet, at the Thornton Heath Infirmary; also a supplementary tender for builder's work.

### OUR BUREAU OF INFORMATION.

### Rules for Correspondents.

1. Every letter, on whatever subject, must be accompanied by the coupon to be cut from the back cover (inside page) of The Hospital, current issue, and must contain the name and address of the correspondent with pseudonym for publication if desired.
2. Replies by post cannot be given, save under exceptional circumstances, at the Editor's discretion.
3. Letters from Approved Homes in reply to special needs published in the Bureau should state terms and full particulars, and be sent prepaid under cover to the Editor of the Bureau with coupon and name enclosed for identification.
4. Proprietors of homes which have not yet been entered on the List of Approved Homes, but have spare accommodation likely to suit special needs, are invited to write for an application form for registration. The fee for registration is 5s.
5. Special needs of every description relating to those who are sick or in difficulties are made known in these columns free of charge, but applications relating to business or employment from correspondents in an independent position must be accompanied by a postal order for 5s.
6. All communications to be addressed to The Editor of The Hospital Bureau of Information, 28 Southampton Street, Strand, London, W.C.

### Needs and Helpers.

### Home for Boy with Tuberculosis of Brain.

"Desirée."—We note you require home for boy of ten with serious eye trouble caused by above disease, and that meningitis is threatened unless immediate treatment can be secured. As the doctor states there is good prospect of recovery if good food and nursing can be procured at once, we would suggest application to the Convalescent Home for Children, Filey Road, Scarborough, where children are received for long periods at a charge of 2s. 6d. a week in winter and 3s. 6d. in summer. Probably the parents could pay half this sum if the other half could be promised. If no payment whatever can be made, the best course to pursue would be to consult the Guardians, and get an order from the relieving officer to take him into the infirmary or sick wards of the workhouse.

### Country Problems.

### Home for Mild Epileptic.

"Mrs. F."—The lowest terms at which epileptics are received into any home are 10s. a week, and I gather from what you say that this is a prohibitive charge in this case. As matters now stand, there is nothing to be done with an epileptic young woman but to keep her at home and provide her with some form of light work which will help to keep her out of mischief. There is a strong call for the provision of colony treatment for sufferers of this class, for they commonly do far better among strangers and under mild discipline than at home. If a little money could be collected it would be helpful to have her trained to do some distinctive kind of work which she could get interested in, so as to implant self-respect. Lace-work is good, as it can be done out of doors, but it is not very remunerative.

### Incipient Consumption in Boy of Ten.

"Walter."—The proportion of children in the elementary schools showing early symptoms of phthisis with high temperature is very large, and your difficulty is being widely felt. The best that can be done is to provide a separate little bed to ensure that the child sleeps alone; to have the window in the room made to open wide; to persuade the mother that his life depends on his getting plenty of fresh air; to secure the aid of the village schoolmaster, to give breathing and other exercises; and, lastly, to send him twice a day to the nurse's house to drink slowly a glass of milk. The recuperative powers of childhood are so great that even a trifling change in the habits and environment may serve to check the disease.

### Hospital Appliances.

### Fire Extinguishers for Cottage Hospital.

"Secretary, F. W."—The patent appliances are only likely to be of service if used in the beginning of the outbreak. Everything therefore depends on having them available all over the house. The least expensive to scatter freely about are the Harden Star Hand Fire Grenades, and these are perhaps the most commonly used. They cost 32s. a dozen, and are to be thrown into the fire whenever it occurs. Wire baskets to hold a couple should be placed in every ward and room, and in different places on the staircases and hall and in the offices. The same makers have also a hand sprinkler at 40s. a dozen. The "Kyl-Fyre" is an efficient dry powder fire-extinguisher very efficacious in putting out small fires. The "New Era" is rather more expensive, but is a good apparatus for hospitals. Whatever appliance is used, it is advisable to give a demonstration to the staff of its effect on a fire kindled in the open from time to time, so that there may be no delay in applying it to its right purpose when occasion requires.

### Conditions of Service.

### Pension of Mental Hospital Worker.

"Mental."—You cannot ensure a pension if you become nurse in a general hospital, but if you enter a mental hospital you will be entitled to a pension equal to one-fiftieth of the total value of your salary and emoluments for each year of service. The most you can receive is two-thirds of the salary and emoluments you have been receiving. You are mistaken in thinking you would have to remain in one institution the whole time in order to qualify for a retiring allowance. But periods of less than two years in one mental hospital will not count.

### Approved Homes.

### For Children Requiring Special Care.

We have pleasure in announcing that we have recently added to our List a thoroughly well-managed Home of a somewhat unusual character for a limited number of children who are in some degree below normal, whether mental or physically. The Home is under the direct supervision of a well-known surgeon, and curative treatment through massage, physical exercises and games, with genial family life, forms a prominent feature. Great care is taken in the selection of children so that there shall be no association with those likely to be hurtful as examples. As publicity is impossible with this class of work, we are requested not to publish name and address, but we are ready to forward letters from any requiring this class of accommodation if addressed to D. I.. Z14, under cover to the Editor of the Bureau.

### List of Homes Wanted.

Nurse Bottomley.—The List is in process of formation and particulars of new Homes added from time to time are published in the Bureau. We shall be pleased at any time to place you in communication, as you desire, with "genuine approved Homes either for people of limited means or for persons able to pay," and if you will send on their requirements to us with pseudonym we will make them known in these columns or send you the name of Homes in the required locality.

### Letter-Box.

W. J. Hannon.—Thanks for your letter. The leaflet will be ready shortly and shall be sent on to you. We are glad to have your co-operation.

Sister Eunice.—Please send coupon.

### Books Received.

Harrison and Sons: "An Operating Theatre in Private Practice," by C. Hamilton Whitefoot.

J. Wright and Sons: "Public Health Chemistry and Bacteriology," by David McKail; "Sprue: Its Diagnosis and Treatment," by Chas. Begg; "Pulmonary Phthisis," by A. Harris.

Sherratt and Hughes: "Diseases of the Nervous System," by J. S. Bury, M.D.

"Chemist and Druggist": "The Art of Dispensing" (ninth edition), by Peter MacEwan.

Eyre and Spottiswoode: "Printers' Pie, 1912."

G. Allen and Co.: "Words to Wives," by Dr. S. Bingham.

Cassell and Co.: "Clinical Methods," by Hutchison and Rainy. Fifth edition.

Lippincott and Co.: "Diseases of Children," by Shaw and Lafeta.

J. A. Churchill and Sons: "System of Treatment," by Latham and English. In four volumes, I. to IV. inclusive.

G. Gill and Sons: "First Aid in Accidents," by Collie and Wightman. Sixth edition.

Methuen and Co., Ltd.: "The Doctor and the People," by H. De Carle Woodcock.

### Appointments.

THE following appointments have been made at St. Mary's Hospital:—Dr. W. J. Gow to be obstetric surgeon, and Dr. T. G. Stevens to be obstetric surgeon in charge of out-patients.

THE following appointments have been made at the Royal Free Hospital:—Mr. J. H. Lawry, M.B., to be house physician; Miss Powell, M.B., to be junior obstetric assistant; Miss Bernard, L.S.A., M.A., to be clinical assistant to radiographer; Miss Bentham, L.R.C.P., to be clinical assistant throat department; Mrs. Addison, M.B., to be clinical assistant skin department; Miss Turnbull, M.D. B.S., to be clinical assistant gynæcological department; and Miss Rathbone, L.S.A., to be clinical assistant to Mr. Cunning.

MR. D. G. RICE-OXLEY, M.B., B.S., has been appointed assistant anæsthetist at the Cancer Hospital in place of Dr. E. V. Dunkley, who has resigned.

### The Week's Novelties.

### PLASMON BISCUITS.

IN these days of quick lunches it is not only travellers who demand concentrated foods. Of those at present in vogue Plasmon holds a high place, which it has attained by force of merit, and further testimony need hardly be added to that of numerous leading medical practitioners who have so frequently affirmed its nutritious virtues. A very convenient form in which Plasmon may be obtained is the Plasmon biscuits. These are made in four varieties, of which perhaps the palm must be given the sweet and the wholemeal. The other two kinds are the plain and the ginger biscuits. These biscuits are exceedingly palatable, and keep in good condition for a reasonably long time. They contain an amount of Plasmon equal to one-fifth of the farinaceous material. The makers are Jacob and Co., Ltd., Dublin.

### Gifts and Bequests.

THE London Homœopathic Hospital has received a promise of £500 from Mr. Edwin Tate towards the extension of the Homœopathic Convalescent Home at Eastbourne for men patients, as well as for women and children as at present. About £3,000 will be required, and Lord Dysart has promised £100 if the balance of £300 still required is given so that the Home can be extended before the coming summer.

THE KING has sent from Buckingham Palace, through the Master of the Household, a gift of linen to the Royal Hospital for Incurables, Putney Heath, of which institution his Majesty is patron, and also a present of cast linen to the Chelsea Hospital for Women.

### THE LEAGUE OF MERCY MATINEE.

THE League of Mercy acknowledges the receipt from His Serene Highness Prince Alexander of Teck—Grand President of the League of Mercy—of the amount of £1,017 17s. 3d. on behalf of the League of Mercy, being the profits obtained by a matinée held at the London Opera House on April 29, which was kindly organised by Dora, Countess of Chesterfield, who is Lady-President of the South Kensington District of the League, and a committee of ladies and gentlemen working with her.

